# Comparison of the ABC/2 formula with computer-assisted volumetry of ischemic cerebellar stroke

**DOI:** 10.1371/journal.pone.0331296

**Published:** 2025-08-26

**Authors:** Milos Arsenovic, Artem Rafaelian, Daniel Dubinski, Daniel Cantré, Eva Herrmann, Bedjan Behmanesh, Svorad Trnovec, Thomas M. Freiman, Marc-André Weber, Matthias Wittstock, Florian Gessler, Sae-Yeon Won

**Affiliations:** 1 Department of Neurosurgery, Rostock University Medical Center, Rostock, Germany; 2 Department of Neuroradiology, Rostock University Medical Center, Rostock, Germany; 3 Institute of Biostatistics, Goethe University Frankfurt, Frankfurt, Germany; 4 Department of Neurology, Rostock University Medical Center, Rostock, Germany; UCSF: University of California San Francisco, UNITED STATES OF AMERICA

## Abstract

**Introduction:**

Current guidelines suggest surgical decompression for ischemic cerebellar stroke in case of significant mass effect. Recent research has aimed to identify a possible threshold for mass effect. However, a computer-assisted volumetry in acute setting is time consuming and impracticable, wherefore the aim of this study was to assess the accuracy and clinical applicability of the ABC/2 method in case of ischemic cerebellar stroke.

**Materials and methods:**

Imaging data of 125 patients, including preoperative CT or MRI scans were used for volumetric analysis. The ABC/2 formula using scans in axial and coronal planes. BrainLab® Elements software was used for computer assisted volumetry by defining the region of interest allowing automated volumetric calculation. Measurements were conducted independently by blinded clinicians. Pearson correlation and Bland-Altmann test were used for statistical analysis.

**Results:**

Among the 125 cerebellar infarctions analyzed, there was no statistical difference of mean infarct volume measurement between the ABC/2 formula and computer-assisted volumetry (16.6mL vs. 15.91mL; range 0.8-67.7mL; p = 0.76). The Spearman correlation test indicated a strong correlation between the two methods (r = 0.985, 95% CI: 0.979–0.990, p < 0.0001). Discrepancies were most notable in smaller infarction volumes (<10 mL), prompting a subgroup analysis. For infarct volume less than 10mL, the ratio of volumetric differences ranged from 47% to 60%, with absolute volume differences from −3–3 mL whereas the ratio ranged from −20% to 29%, with absolute volume differences from −6–8 mL in cases with infarct volume greater equal 10mL.

**Conclusion:**

ABC/2 formula shows a good correlation with computer-assisted volumetry. Consequently, it could serve as a fast and practical tool for estimating cerebellar infarct volume and aiding decision-making in clinical practice. However, the limitations and variability of the ABC/2 method, particularly for smaller infarcts, must be considered.

## Introduction

Surgical treatment of ischemic cerebellar stroke is recommended according to the current American Heart Association/American Stroke Association (AHA/ASA) and European Stroke Organisation (ESO) guidelines in cases of neurological deterioration or presence of a mass effect [1,2]. Recently, our group has shown in a large cohort of patients with ischemic cerebellar infarcts that a subgroup of patients with infarct volume of 35mL or greater display improved outcome when treated surgically, while conservative treatment was better in patients with cerebellar infarct volume less than 25mL [3]. Since then, volumetric threshold has been a helpful decision maker to indicate surgical or conservative treatment.

In clinical setting, computer-assisted volumetry is considered as the gold standard. Otherwise, simplified volumetric formula is easily applicable and practicable with a possible loss of accuracy. Previously, the ABC/2 formula has been introduced to measure volumes of pathologies including intracerebral haemorrhage, vestibularis schwannoma, subdural hematoma, pneumocephalus and subarachnoid hemorrhage showing a good correlation to computer-assisted volumetry [4–10]. However, the formula has not yet been proven in case of infarct volumetry. The aim of this study was to evaluate the accuracy of the ABC/2 method on its clinical applicability.

## Materials and methods

The study was approved by the local ethics committee at the Rostock University Medical Center (registration no. A2020-0266). Informed consent of patients was waived given the blinded, retrospective nature of the study.

In this study, all patients with ischemic cerebellar infarction admitted in the authors’ institution from August 2016 to March 2024 were included. Patients with insufficient radiological data (e.g. slice thickness over 3 mm), concomitant stroke in the supratentorial compartment, stroke with hemorrhagic transformation or sole brainstem infarction were excluded resulting in a total of 125 patients for the final analysis. The data were assessed from 1^st^ July 2024 until 15^th^ December 2024.

The preoperative CT- and MRI-scans were used for computer-assisted measurement and ABC/2 with standardized protocols (CT: GE revolution CT, topogram, collimation with 256 slices each 0.625 mm thickness, native head scan with reconstruction using bone and soft-tissue kernels; MRI: 1.5 T or 3 T, EPI DWI, T2 FLAIR, T1-weighted, T2-weighted, 3D TOF with MIP reconstruction, 3D T1 MPRAGE with multiplanar reconstruction (MPR), T1 TSE fat-saturated sequences in both axial and coronal planes). Each method was used by an independent clinician in a blinded fashion.

**Fig 1:**
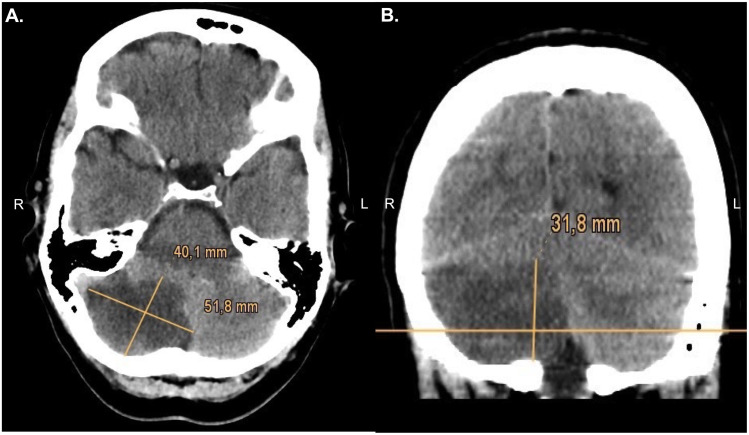
Volumetric measurement of cerebellar infarct tissue via ABC/2 formula resulting in a infarct volume of 33.3 mlA. A defined as the largest diameter of cerebellar infarction in the axial slice: 5.18 cm; B defined as the maximum length 90° to A: 4.01 cm B. C as the maximum height of the cerebellar infarction in coronar plane: 3.18 cm.

For the ABC/2 method, the largest diameter of cerebellar infarction was defined as A (cm), the maximum width (lateral to midline) 90° to A in the same axial slice as B (cm) and the maximum height of the cerebellar infarction C (cm) in the coronal plane. The volume was calculated by multiplying A, B and C and dividing it by 2 [4] (Fig 1).

**Fig 2:**
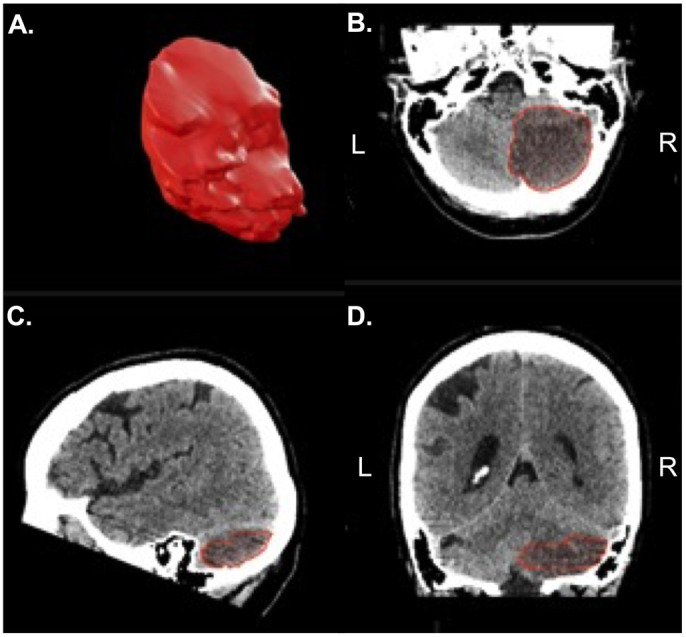
Computer-assisted semi-automatic volumetry via Brainlab for the same patient resulting in an infarct volume of 35.6mL. A. 3D model reconstruction of infarct tissue. Region of interest in the axial (B.), sagittal (C.) and coronar (D.) plane.

For the computer-assisted measurement, the BrainLab® elements software (Brainlab Germany Headquarters, Munich, Germany) was used. The region of interest (cerebellar infarct) was defined and the volume was automatically calculated (Fig 2).

### Statistical analysis

After obtaining the volume (mL) by the ABC/2 formula and computer-assisted analysis, linear regression analysis of Spearman and Bland-Altmann regression analysis were performed to determine the correlation and range of discrepancy between the two methods. The Kolmogorov-Smirnov test revealed non-normal distribution of the volumetric data sets wherefore Mann-Whitney-U test was applied in the statistical analysis. A p-value of ≤ 0.05 was assumed as statistically significant. For the analysis, Graphpad version 9.4.2. was used.

## Results

In total, 125 cerebellar infarctions were included in the final analysis. The mean age was 70.9 ± 7.4 years and 80 patients (64%) were female. The median GCS and NIHSS at admission was 15 and 4, respectively. Among them, 24 patients (19.2%) underwent thrombolysis, 13 patients (10.4%) mechanical recanalization and 14 patients (11.2%) surgical decompression due to mass effect (S1 Table).

There was no significant difference of mean infarct volumes using the ABC/2 formula versus computer-assisted volumetry (16.6mL ± 16.9 vs. 15.9mL ± 16.4; p = 0.76). The calculated volumes ranged from 0.79 mL to 67.6 mL, with 15 infarct volumes exceeding 35mL.

**Fig 3:**
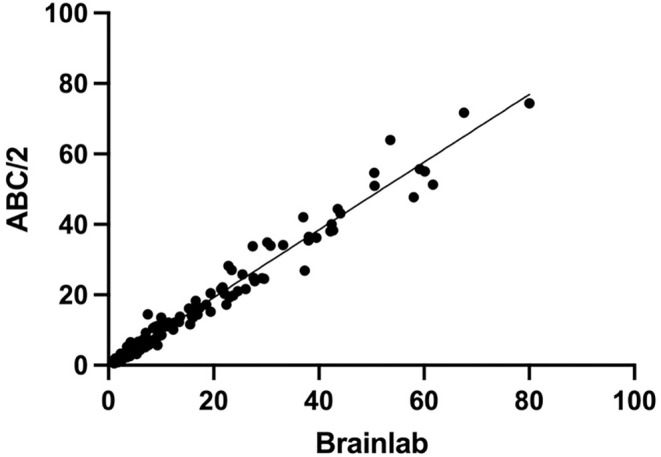
Spearman correlation test between ABC/2 and computer-assisted volumetry (in ml) showing an excellent correlation (r = 0.985, CI 95% 0.979-0.990; p value <0.0001).

**Fig 4:**
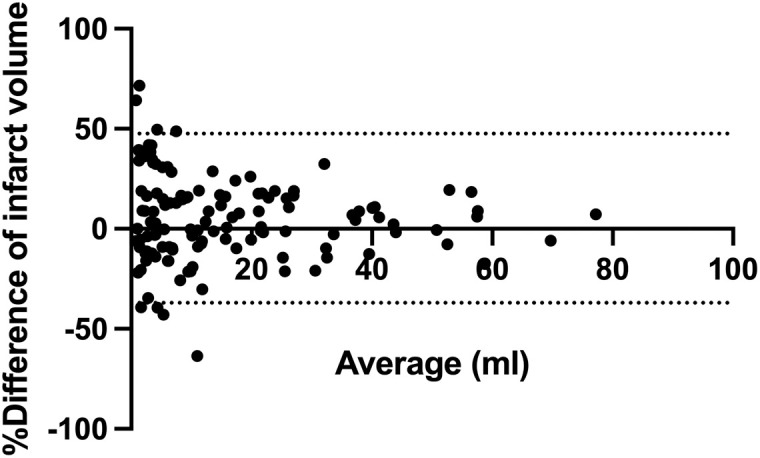
Bland-Altmann-analysis with range of discrepancy in ratio (%) between ABC/2 formula and computer-assisted volumetry.

In the Spearman correlation test, the both methods showed an excellent correlation (r = 0.985, CI 95% 0.979–0.990; p value <0.0001) (Fig 3). The Bland-Altman analysis showed a mean volume difference (bias) of −5.3 ml ± 21.6 with limits of agreement (LoA) ranging from −37% to 48% between ABC/2 and computer-assisted volumetry suggesting clinically significant variability between the two methods (Fig 4).

Of note, the ratio showed significant discrepancy regarding lower volume of cerebellar infarct (<10ml). We therefore divided the full cohort into subgroups: patients with 10mL or greater infarct volume versus patients with less than 10mL infarct volume. The ratio of volume difference in patients with less than 10mL was significantly higher ranging from −47% to 60% which had an absolute volumetric range between −3 to 3mL. On the other hand, patients with 10mL or greater volume showed significantly reduced volumetric discrepancy with a ratio between −20 to 29%, which accounted an absolute range of −6 to 8mL (S1 Fig).

## Discussion

Previously, the ABC/2 formula has been shown as a precise and valuable tool for simplified and rapid volumetric analysis in a wide range of pathologies [4–10]. In this study, we demonstrated an excellent correlation of the ABC/2 formula with computer-assisted measurement of ischemic cerebellar infarct volume as well which widens the indication for the safe use of the ABC/2 formula.

Of note, the precision of the simplified formula might depend on the slice thickness of CT or MRI-scan since small volumes with small length/wide/height could be missed in a thicker axial or coronal plane leading to volumetric under- or overestimation [11,12]. This effect could be observed in our cohort. Patients with infarct volume less than 10mL demonstrated a wider range of discrepancy compared to those with larger infarct volume. Although this may have no potential impact on clinical decision-making due to its minor mass effect, such observation should be taken into account.

Further, patients with an infarct volume of greater than 10mL had a discrepancy of volumes ranging from −20% to 29%. In a clinically relevant application, our research group previously analyzed the threshold of mass effect to treat cerebellar infarction surgically and found out a lesion volume of 35mL or above as suitable [3]. Similar result with 38 ml as a significant factor for the development of malignant cerebellar edema was reported by a recent study from Baki et al. [13] When applying the results obtained within the current studies by measuring the lesion volume via ABC/2 formula, a range of 28-45mL should be considered as relevant mass effect warranting surgery. However, minor discrepancies in volumetric estimation should be considered clinically insignificant, and treatment should be primarily guided by the patient´s neurological status. The volumetric assessment should be incorporated as a supporting factor in the overall management strategy.

To date, there are several alternative methods for volumetric estimation via manual segmentation with automatic volumetry or AI-based volumetry particularly for the diagnostic of neurodegenerative disease [14,15]. Despite its high accuracy and automation, manual segmentation is time-consuming and operator-dependent as well as AI-software often requires technical infrastructure and validation. In our personal view, ABC/2 is quick, reproducible and easy to apply using standard imaging for all clinicians in low- as high-income countries. Its simplicity and accessibility make it preferable in urgent care scenarios, particularly when rapid surgical decision-making is required and advanced tools are not immediately available.

There are some limitations to mention. First, the differentiation between infarct tissue and edema was in a CT-scan not possible; the whole hypodense area was assumed as cerebellar infarct area. That might have led to overestimation of the infarct volume, however, since the computer-assisted volumetry was performed by marking the same region of interest, both volumetric methods would have no gradual difference in the final analysis. Second, some of the cerebellar infarcts were multilocular which made it necessary to measure and add each measurement of ABC/2. That might have resulted in a cumulation of volumetric discrepancy. Despite these limitations, the analysis of both methods revealed an excellent correlation. Third, this study’s retrospective, single-center design restricts the generalizability of its findings. Without external validation using independent datasets, caution is needed when applying these results to other institutions or populations. Future prospective, multicenter studies are necessary to validate the accuracy and clinical utility of the ABC/2 formula across diverse settings.

## Conclusion

ABC/2 is a precise and formula allowing for adequate measurement of lesion volumes in patients with ischemic cerebellar stroke. The results obtained may ease the decision making for surgical treatment. If used, a possible range of discrepancy of 20% should be considered in the clinical practice.

## Supporting information

S1 Table Supplementary Table S1. (DOCX)

S1 Fig Supplementary Fig S1. (TIFF)

S2 Minimal data set can be provided upon request. (XLSX)

## References

[1] van der Worp HB, Hofmeijer J, Jüttler E, Lal A, Michel P, Santalucia P, et al. European Stroke Organisation (ESO) guidelines on the management of space-occupying brain infarction. Eur Stroke J. 2021;6(2):XC–CX. 34414308 10.1177/23969873211014112 PMC8370072

[2] Powers WJ, Rabinstein AA, Ackerson T, Adeoye OM, Bambakidis NC, Becker K, et al. Guidelines for the Early Management of Patients With Acute Ischemic Stroke: 2019 Update to the 2018 Guidelines for the Early Management of Acute Ischemic Stroke: A Guideline for Healthcare Professionals From the American Heart Association/American Stroke Association. Stroke. 2019;50(12):e344–418. doi: 10.1161/STR.0000000000000211 31662037

[3] Won SY, Hernández-Durán S, Behmanesh B, Bernstock JD, Czabanka M, Dinc N. Functional Outcomes in Conservatively vs Surgically Treated Cerebellar Infarcts. JAMA Neurology. 2024;81(4):384–93. 38407889 10.1001/jamaneurol.2023.5773 PMC10897822

[4] Kothari RU, Brott T, Broderick JP, Barsan WG, Sauerbeck LR, Zuccarello M, et al. The ABCs of measuring intracerebral hemorrhage volumes. Stroke. 1996;27(8):1304–5. doi: 10.1161/01.str.27.8.1304 8711791

[5] Won S-Y, Zagorcic A, Dubinski D, Quick-Weller J, Herrmann E, Seifert V, et al. Excellent accuracy of ABC/2 volume formula compared to computer-assisted volumetric analysis of subdural hematomas. PLoS One. 2018;13(6):e0199809. doi: 10.1371/journal.pone.0199809 29944717 PMC6019668

[6] Bathla G, Policeni B, Hansen MR, Berbaum K. Calculating the Tumor Volumes in Vestibular Schwannomas: Are the ABC/2 and Volumetric Methods Comparable? Otol Neurotol. 2017;38(6):889–94. doi: 10.1097/MAO.0000000000001423 28394785

[7] Föttinger F, Sharma R, Salman SD, Weston AD, Erickson BJ, Huynh T. The ABCs of Subarachnoid Hemorrhage Blood Volume Measurement: A Simplified Quantitative Method Predicts Outcomes and Delayed Cerebral Ischemia. J Am Heart Assoc. 2024;13(20). 10.1161/JAHA.123.032195 PMC11935569 39392139

[8] Fletcher-Sandersjöö A, Lewén A, Hånell A, Nelson DW, Maegele M, Svensson M. Volumetric assessment of traumatic intracranial hematomas: Is ABC/2 reliable? Journal of Neurotrauma. 2024;41(23–24):2545–53. 39162998 10.1089/neu.2024.0248

[9] Chan DYC, Cheung EYH, Hui KH, Leung CMS, Ng SCP, Mak WK, et al. ABC/2 formula for “bedside” postoperative pneumocephalus volume measurement. Chin Neurosurg J. 2022;8(1):18. 35922864 10.1186/s41016-022-00287-z PMC9347102

[10] Tanioka S, Aydin OU, Hilbert A, Kitano Y, Ishida F, Tsuda K. Reliability of ABC/2 volumetric estimation in spontaneous intracerebral hemorrhage for hematoma expansion prediction scores. Eur Stroke J. 2024. 10.1177/23969873241293572 PMC11556596 39474681

[11] Delcourt C, Carcel C, Zheng D, Sato S, Arima H, Bhaskar S, et al. Comparison of ABC Methods with Computerized Estimates of Intracerebral Hemorrhage Volume: The INTERACT2 Study. Cerebrovasc Dis Extra. 2019;9(3):148–54. doi: 10.1159/000504531 31838472 PMC6940457

[12] Maeda AK, Aguiar LR, Martins C, Bichinho GL, Gariba MA. Hematoma volumes of spontaneous intracerebral hemorrhage: the ellipse (ABC/2) method yielded volumes smaller than those measured using the planimetric method. Arq Neuropsiquiatr. 2013;71(8):540–4. doi: 10.1590/0004-282X20130084 23982013

[13] Baki E, Baumgart L, Kehl V, Hess F, Wolff AW, Wagner A. Predictors of malignant swelling in space-occupying cerebellar infarction. Stroke Vasc Neurol. 2024. 10.1136/svn-2024-003360 PMC12230214 39209704

[14] Rudolph J, Rueckel J, Döpfert J, Ling WX, Opalka J, Brem C, et al. Artificial intelligence–based rapid brain volumetry substantially improves differential diagnosis in dementia. Alzheimer’s and Dementia: Diagnosis, Assessment and Disease Monitoring. 2024;16(4). 10.1002/dad2.70037 PMC11632536 39665087

[15] Purrer V, Pohl E, Lueckel JM, Borger V, Sauer M, Radbruch A, et al. Artificial-intelligence-based MRI brain volumetry in patients with essential tremor and tremor-dominant Parkinson’s disease. Brain Commun. 2023;5(6):fcad271. doi: 10.1093/braincomms/fcad271 37946794 PMC10631860

