# Supplementary material for: Comparison of the ABC/2 formula with computer-assisted volumetry of ischemic cerebellar stroke
Source: PLoS One. 2025 Aug 26;20(8):e0331296. doi: 10.1371/journal.pone.0331296 (PMC12380265; doi:10.1371/journal.pone.0331296)
Supplement: S1 Table — (DOCX) [file pone.0331296.s001.docx]

**Table 1.** Baseline characteristics of patients with ischemic cerebellar infarction.

| **Variables** | n=125 |
| --- | --- |
| Age, mean (SD) | 70.9 ± 7.4 |
| Sex (female) | 80 (64%) |
| GCS at admission, median (IQR) | 15 (14-15) |
| NIHSS at admission, median (IQR) | 4 (1-6.5) |
| NIHSS at discharge, median(IQR) | 2 (0-4.5) |
| mRS at discharge, median (IQR) | 2 (1-3) |
| mRS at 12 months, median (IQR) | 2 (0-4) |
| Hypertension | 99 (79.2%) |
| Diabetes mellitus type II | 33 (26.4%) |
| COPD/Asthma | 12 (9.6%) |
| Anticoagulation | 14 (11.2%) |
| Atrial fibrilation | 30 (24 %) |
| Prior ischemic stroke | 30 (24 %) |
| Prior antiplatelet use | 36 (28.8%) |
| Coronary artery disease | 23 (18.4 %) |
| Thrombolysis | 24 (19.2%) |
| Mechanical recanalization | 13 (10.4%) |
| Surgical | 14 (11.2%) |
| Pneumonia | 20 (16%) |
| Urinary tract infection | 15 (12%) |

SD, standard deviation; GCS, Glasgow coma scale; IQR, Interquartile Range; NIHSS, The National Institutes of Health Stroke Scale; mRS, modified Ranking Scale;
