# Supplementary figures and images for: Comparison of the ABC/2 formula with computer-assisted volumetry of ischemic cerebellar stroke
Source: PLoS One. 2025 Aug 26;20(8):e0331296. doi: 10.1371/journal.pone.0331296 (PMC12380265; doi:10.1371/journal.pone.0331296)

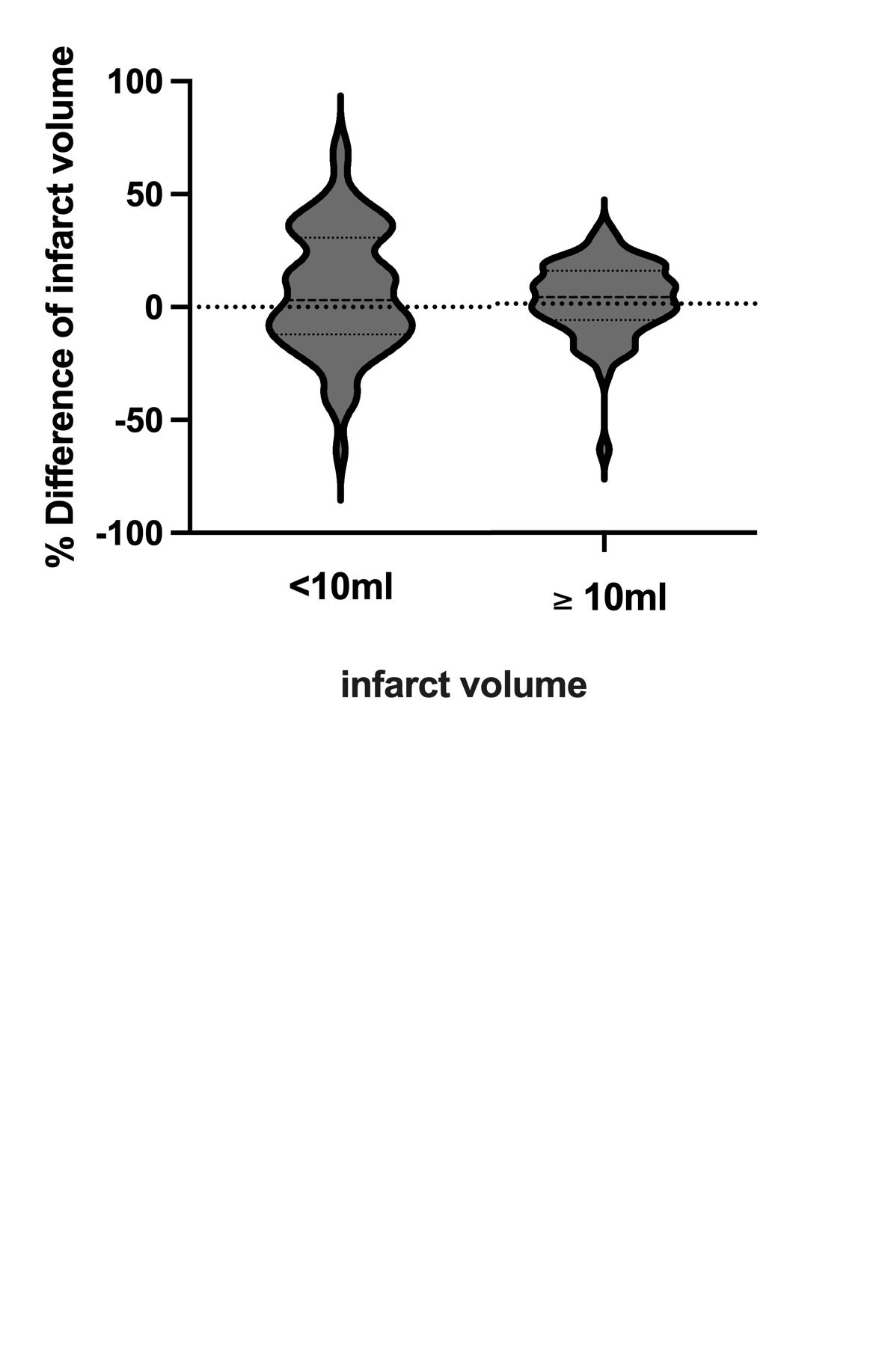

Supplement: S1 Fig — (TIFF) [file pone.0331296.s002.tiff]
